# Toward evidence-based fish welfare regulation: policy lessons from Chile, Norway, and Scotland

**DOI:** 10.3389/fvets.2026.1972966

**Published:** 2026-10-23

**Authors:** Juan Antonio Valdés

**Affiliations:** 1 Laboratorio de Biotecnología Molecular, Facultad de Ciencias de la Vida, Universidad Andres Bello, Santiago, Chile; 2 Interdisciplinary Center for Aquaculture Research—Applied Research (INCAR^2^), Universidad Andres Bello, Santiago, Chile; 3 Centro de Investigación Marina Quintay (CIMARQ), Universidad Andres Bello, Quintay, Chile

**Keywords:** aquaculture, Evidence-Based Welfare Regulation, fish welfare, Operational welfare indicators, stress

## Abstract

Aquaculture has become a major contributor to global food production, but its rapid expansion and intensification have increased concerns regarding the welfare of farmed fish. Although advances in welfare science have generated animal-based measures, operational welfare indicators, and increasingly sophisticated monitoring technologies, their incorporation into regulatory frameworks remains uneven. This review examines the evolution of fish welfare regulation and compares the regulatory approaches of Chile, Norway, and Scotland, three major salmon-producing jurisdictions representing distinct models of welfare governance. The analysis distinguishes between legislation that directly establishes welfare obligations and sanitary, environmental, biosecurity, and production regulations that indirectly influence welfare determinants. Chile combines explicit legal recognition of fish welfare with an extensive sanitary and environmental regulatory architecture, although standardized animal-based welfare assessment, intervention criteria, and welfare-specific auditing remain limited. Norway is characterized by the direct incorporation of detailed welfare requirements into aquaculture regulation, complemented by structured monitoring, documentation, and science-based assessment tools. Scotland represents a more distributed model in which statutory welfare duties are complemented by aquaculture and animal-health legislation, government guidance, industry codes, and scientific assessment tools. Importantly, differences in regulatory operationalization should not be interpreted as evidence of differences in regulatory effectiveness, which requires independent evaluation through measurable welfare outcomes. Based on this comparison, we propose Evidence-Based Welfare Regulation (EBWR) as an integrative regulatory framework connecting legal welfare objectives with evidence-qualified resource-, management-, and animal-based indicators, standardized monitoring, context-specific thresholds or reference ranges, corrective actions, outcome verification, and periodic scientific review. Rather than introducing a new welfare-assessment methodology, EBWR integrates existing scientific and regulatory tools within an adaptive feedback system. For Chile, this transition could build on existing regulatory and institutional capacities through species-specific welfare standards, evidence-qualified operational welfare indicators, risk-based monitoring, outcome verification, and adaptive regulatory review.

## 1 Introduction

Aquaculture has become the fastest-growing food production sector worldwide and is expected to play an increasingly important role in meeting the rising global demand for animal protein while reducing pressure on declining wild fisheries (1). Over the last three decades, continuous technological innovation, selective breeding, nutritional improvements, and advances in aquatic animal health have transformed aquaculture into one of the principal contributors to global food security (2). Today, more than half of all aquatic animals consumed by humans originate from aquaculture, making the sector essential for ensuring sustainable food production in the coming decades (3). However, the rapid expansion and intensification of aquaculture have also raised increasing concerns regarding the welfare of the billions of fish reared annually under commercial conditions (4). Consequently, fish welfare is now widely recognized as a fundamental component of sustainable aquaculture, alongside environmental protection, economic viability, and social responsibility (5). Historically, welfare in aquaculture was interpreted largely from a production-oriented perspective, whereby healthy fish exhibiting rapid growth, efficient feed conversion, and low mortality were assumed to experience acceptable welfare (6, 7). Although this approach proved valuable during the early development of commercial aquaculture, accumulating scientific evidence has demonstrated that productive animals may nevertheless experience chronic stress, pain, or behavioral restriction (8). Poor welfare not only compromises ethical standards but also reduces production efficiency by impairing growth, feed utilization, disease resistance, reproductive performance, flesh quality, and survival. Consequently, fish welfare should no longer be viewed as an additional production cost but rather as a biological prerequisite for sustainable and economically efficient aquaculture (9).

At the same time, advances in neurobiology, physiology, and veterinary science have fundamentally changed our understanding of fish biology (10). Research conducted during the last two decades has demonstrated that many fish species possess sophisticated cognitive abilities, learning capacity, memory, behavioral flexibility, social interactions, and the neurophysiological mechanisms necessary to perceive noxious stimuli and experience negative affective states (11). Although questions remain regarding species-specific differences in sentience and consciousness, there is now broad scientific consensus that fish are sentient vertebrates whose welfare deserves ethical and legal consideration. This recognition has profoundly influenced public attitudes, certification programs, industry practices, and governmental policies worldwide (12). The scientific concept of fish welfare has evolved in parallel with these discoveries. Early welfare frameworks were strongly influenced by the Five Freedoms, emphasizing freedom from hunger, discomfort, pain, disease, fear, and the opportunity to express natural behavior (13). More recently, welfare science has adopted more comprehensive approaches, particularly the Five Domains Model, which integrates nutrition, physical environment, health, behavior, and mental state while recognizing both negative and positive experiences (14). Similarly, the One Welfare concept has highlighted the interdependence between animal welfare, environmental sustainability, ecosystem health, food security, and human wellbeing (15). These conceptual developments have considerably broadened welfare assessment beyond traditional indicators of health and productivity.

Advances in welfare science have also transformed assessment methodologies. Traditional evaluations based primarily on mortality, growth, and disease prevalence are increasingly complemented by Animal-Based Measures (ABMs), Operational Welfare Indicators (OWIs), physiological biomarkers, behavioral observations, health scoring systems, and environmental monitoring (16). More recently, precision aquaculture technologies, including biosensors, computer vision, artificial intelligence, and omics approaches, have opened new opportunities for objective and evidence-based welfare assessment (17). These developments have given rise to the concept of Evidence-Based Animal Welfare, in which management decisions, certification schemes, and regulatory standards are supported by validated scientific evidence rather than solely by tradition or expert opinion (18). Despite these advances, fish welfare legislation has progressed more slowly than welfare science. Many regulations continue to emphasize husbandry, disease control, transport, and slaughter, while providing limited guidance on validated welfare indicators, positive welfare, and emerging monitoring technologies. Consequently, a significant gap remains between current scientific knowledge and the regulatory frameworks intended to protect farmed fish (19).

## 2 Review objective, scope, and analytical approach

Although numerous reviews have addressed fish welfare science, assessment methods, ethics, and regulatory frameworks, comparatively few have examined how advances in welfare science are translated into legally enforceable and operational requirements in aquaculture. Moreover, comparative analyses assessing the extent to which regulatory systems in major salmon-producing jurisdictions reflect contemporary welfare science remain limited. Accordingly, the objective of this narrative review is to compare how fish welfare is incorporated and operationalized within the aquaculture regulatory frameworks of Chile, Norway, and Scotland, and to identify principles that could support the transition toward Evidence-Based Welfare Regulation (EBWR). These jurisdictions were selected because of their substantial contribution to global salmonid aquaculture and because they represent contrasting approaches to welfare governance. Norway and Chile dominate global farmed Atlantic salmon (*Salmo salar*) production, accounting together for approximately 83% of the global total, while Scotland contributes around 7% (20, 21). Thus, the three jurisdictions collectively represented approximately 90% of global farmed Atlantic salmon production, providing a particularly relevant basis for regulatory comparison (22).

A structured narrative search was conducted to identify the scientific literature and regulatory materials included in the review. Peer-reviewed literature was identified through Web of Science and Scopus, using combinations of search terms including “fish welfare,” “aquaculture welfare,” “salmon welfare,” “animal-based indicators,” “operational welfare indicators,” “welfare assessment,” “welfare regulation,” “aquaculture regulation,” “fish welfare legislation,” “salmon aquaculture,” and the jurisdictional terms “Chile,” “Norway,” and “Scotland.” Searches covered publications available up to September 2026, with particular emphasis on recent literature describing contemporary welfare-assessment approaches and regulatory developments. Regulatory and policy documents were identified through official governmental and legislative repositories in each jurisdiction, including the Biblioteca del Congreso Nacional de Chile (BCN) for Chile, Lovdata and official Norwegian Government sources for Norway, and legislation.gov.uk and Scottish Government sources for Scotland. These searches were complemented by international standards and guidance, including relevant World Organization for Animal Health (WOAH) resources, as well as peer-reviewed scientific literature. Documents were included when they contributed directly to at least one of the following dimensions: (i) legal recognition or protection of farmed fish; (ii) aquaculture-specific welfare requirements; (iii) use of resource-, management-, or animal-based welfare indicators; (iv) standardized welfare monitoring; (v) intervention thresholds or criteria triggering management responses; (vi) corrective or preventive actions; (vii) regulatory oversight, auditing, or enforcement; or (viii) mechanisms for incorporating updated scientific evidence into regulatory or management standards.

Sources were prioritized according to their relevance and authority, with primary legislation and regulations considered first, followed by government guidance, international standards, and peer-reviewed scientific literature. Regulatory instruments were classified according to their legal and operational functions into four categories: (i) general animal-welfare legislation; (ii) aquaculture-specific rules that directly establish or operationalize fish-welfare duties; (iii) sanitary, environmental, biosecurity, and production rules with indirect welfare implications; and (iv) non-statutory guidance, industry codes, and scientific welfare-assessment tools. The comparison was qualitative and was not designed to rank national regulatory systems or to assume that greater regulatory detail necessarily results in better welfare outcomes. Instead, the central analytical criterion was the degree of welfare operationalization, defined here as the extent to which broad legal obligations are translated into scientifically supported and measurable welfare indicators or outcomes, standardized monitoring procedures, context-appropriate intervention thresholds, corrective actions, regulatory oversight and enforceability, and mechanisms for periodic scientific review. This distinction is important because formal inclusion of fish within animal-welfare legislation does not necessarily result in aquaculture-specific mechanisms for assessing, verifying, or enforcing welfare outcomes.

## 3 Evolution of fish welfare science

The scientific understanding of fish welfare has undergone a substantial transformation over the past five decades. Historically, fish received limited consideration in animal-welfare science and regulation, partly because their capacity to experience pain, stress, and other affective states was questioned (23, 24). Advances in comparative physiology, neurobiology, ethology, and animal-welfare science progressively challenged this view, demonstrating specialized nociceptive mechanisms, conserved neuroendocrine stress pathways, and complex behavioral responses (25, 26). These developments established fish welfare as a multidisciplinary field integrating physiology, behavior, neuroscience, veterinary medicine, and aquaculture to evaluate the physical and mental state of fish under human care (27).

The conceptual foundations of modern animal-welfare science originated with concerns over intensive livestock production and the development of the Five Freedoms following the Brambell Report (28). Although initially developed for terrestrial animals, these principles influenced subsequent approaches to fish welfare. More recently, the Five Domains Model broadened welfare assessment by integrating nutrition, physical environment, health, behavioral interactions, and mental state, while recognizing that welfare involves both minimizing negative experiences and providing opportunities for positive ones (13, 29). This conceptual evolution has reinforced the need to assess fish welfare through multiple complementary dimensions rather than through individual physiological or production-related variables. In parallel, research on fish stress physiology identified the hypothalamic–pituitary–interrenal (HPI) axis and cortisol as central components of responses to environmental and husbandry challenges (30, 31). Early welfare assessments therefore relied heavily on physiological indicators such as plasma cortisol, glucose, lactate, osmolality, and hematological variables (32). However, the context dependence of these responses and the capacity of fish to physiologically adapt to prolonged challenges demonstrated that no single indicator can adequately characterize welfare. This prompted a shift toward multidimensional approaches integrating physiological, behavioral, health-related, environmental, and production-related information (30, 33, 34). Behavioral responses, health status, injuries, disease, and mortality consequently became increasingly important components of welfare assessment. The development of Animal-Based Measures (ABMs) and Operational Welfare Indicators (OWIs) has further facilitated the translation of welfare science into practical assessment frameworks applicable under commercial aquaculture conditions (34, 35). These approaches complement resource- and management-based indicators by providing information on biological and behavioral responses of fish. However, individual indicators should not be interpreted in isolation, as their welfare relevance may depend on species, life stage, environmental context, and interactions among multiple stressors. Emerging precision-aquaculture technologies may further improve continuous and non-invasive welfare monitoring and facilitate earlier detection of welfare risks (36, 37). Collectively, these scientific advances have provided the basis for incorporating welfare assessment into certification schemes, international guidelines, and national regulatory frameworks. Nevertheless, the availability of scientifically informed welfare indicators does not necessarily ensure their effective regulatory implementation, highlighting the need to examine how welfare science is translated into measurable requirements, monitoring procedures, intervention criteria, corrective actions, and regulatory oversight.

## 4 Evolution of fish welfare legislation

The development of fish welfare legislation has been a gradual process shaped by advances in animal welfare science, increasing societal concern for the ethical treatment of farmed animals, and the expansion and intensification of global aquaculture. Compared with terrestrial livestock, explicit legal protection addressing fish welfare emerged relatively recently (33, 38, 39), partly reflecting historical uncertainty regarding fish cognition, pain perception, and sentience (40, 41). Early legal protection was generally incorporated within broader animal-protection legislation. In the United Kingdom, the Cruelty to Animals Act of 1876 and the Protection of Animals Act of 1911 represented early steps toward the legal protection of fish, although their provisions focused primarily on preventing unnecessary cruelty rather than regulating farming, handling, transport, or slaughter conditions (42–44).

A significant transition occurred during the late 20th century as scientific evidence concerning fish nociception, stress physiology, and behavior challenged previous assumptions regarding fish sentience and contributed to broader recognition of their welfare needs (45, 46). At the European level, the Treaty of Amsterdam formally recognized animals as sentient beings and required animal welfare to be considered in relevant policy areas (47). Directive 98/58/EC subsequently established general welfare principles for animals kept for farming purposes, including fish, requiring measures to ensure welfare and prevent unnecessary pain, suffering, or injury (48). Although these instruments did not establish comprehensive fish-specific requirements, they contributed to a shift from legislation focused predominantly on preventing cruelty toward broader responsibilities for welfare during routine husbandry. Scientific evidence subsequently became increasingly influential in the development of fish-welfare standards. The European Food Safety Authority (EFSA) issued scientific opinions addressing welfare during transport, slaughter, and husbandry (49–52), while the World Organization for Animal Health (WOAH) developed international standards covering transport, stunning, slaughter, disease control, and emergency killing (53). In parallel, private certification schemes emerged as an additional component of aquaculture welfare governance (54). Programs such as the Aquaculture Stewardship Council (ASC), GLOBALG.A.P., Best Aquaculture Practices (BAP), and RSPCA Assured incorporated welfare-related requirements concerning environmental conditions, health management, mortality, stocking density, water quality, handling, and slaughter (18, 55). Together, these developments illustrate the increasing incorporation of scientific evidence and measurable welfare-related criteria into aquaculture governance.

Nevertheless, the translation of welfare science into regulation remains heterogeneous among major aquaculture-producing jurisdictions. Norway, for example, explicitly incorporates fish within its Animal Welfare Act and complements these general obligations with aquaculture-specific requirements addressing husbandry, transport, slaughter, environmental conditions, health, and welfare monitoring (56–59). Chile has developed an extensive regulatory framework addressing biosecurity, aquatic animal health, environmental monitoring, and production management, although explicit provisions concerning animal-based welfare assessment and standardized welfare auditing remain more limited (60, 61). These differences reflect contrasting degrees and mechanisms of welfare operationalization but should not, by themselves, be interpreted as evidence of differences in actual welfare outcomes. Regulatory operationalization and regulatory effectiveness therefore need to be considered as distinct dimensions. More recently, welfare governance has increasingly incorporated Animal-Based Measures (ABMs), Operational Welfare Indicators (OWIs), physiological and behavioral measures, and monitoring technologies alongside traditional resource- and management-based indicators (19). However, the incorporation of welfare indicators into regulatory frameworks does not itself guarantee effective welfare protection. Their regulatory application requires consideration of scientific validity, species and life-stage specificity, environmental context, interactions among stressors, and the strength of evidence supporting intervention thresholds. This progression provides the basis for Evidence-Based Welfare Regulation (EBWR), in which scientifically supported welfare indicators are linked to context-appropriate intervention criteria, corrective actions, outcome verification, regulatory oversight, and periodic scientific review.

## 5 Comparative analysis of fish welfare legislation

### 5.1 Fish welfare legislation in Chile

Chile is the world's second-largest producer of farmed salmon and one of the leading aquaculture-producing nations globally (20, 21, 62). Consequently, the country has developed an extensive regulatory framework governing aquaculture, primarily focused on environmental sustainability, aquatic animal health, biosecurity, disease control, and production management (63). Although many of these regulatory instruments have important implications for fish welfare, welfare has historically been incorporated mainly through sanitary, environmental, and production requirements rather than through a comprehensive regulatory framework based on direct assessment of animal outcomes.

The cornerstone of Chilean aquaculture legislation is the General Fisheries and Aquaculture Law (Ley General de Pesca y Acuicultura, LGPA; Law No. 18,892), complemented by the Environmental Regulation for Aquaculture (RAMA), the Regulation for the Protection, Control and Eradication of High-Risk Diseases in Aquatic Species (RESA), and the Hydrobiological Pest Regulation (REPLA) (64–67). Additional instruments establish requirements concerning stocking density, environmental monitoring, disease surveillance, biosecurity, mortality reporting, sanitary treatments, and movement of aquatic animals (68). Although these instruments were not primarily conceived as welfare regulations, they influence important welfare determinants by regulating environmental, sanitary, and husbandry conditions. Fish welfare is also explicitly recognized within the LGPA. Article 13F establishes that aquaculture must include provisions safeguarding animal welfare and procedures intended to avoid unnecessary suffering (64). However, it does not define fish welfare or establish a standardized framework of Animal-Based Measures (ABMs) or Operational Welfare Indicators (OWIs) through which welfare outcomes can be systematically assessed at farm level. A more specific provision is found in Article 32B of the RESA, which requires fish to be sedated or rendered insensible before gill cutting to avoid unnecessary suffering (66). Nevertheless, this requirement remains predominantly procedure-based because standardized animal-based criteria for verifying effective stunning and maintenance of unconsciousness until death are not specified.

The broader legal context is provided by the Animal Protection Law (Law No. 20,380), which establishes general principles concerning appropriate animal treatment and prevention of unnecessary suffering and applies subsidiarily to matters governed by the LGPA (68). Chile therefore combines explicit welfare provisions with a broader set of sanitary, environmental, and production regulations that indirectly influence welfare. Importantly, the existing system already generates substantial information potentially relevant to welfare, including mortality, disease occurrence, stocking density, sanitary treatments, and environmental conditions. However, these data are primarily interpreted within sanitary, environmental, epidemiological, or production-control frameworks rather than integrated into a standardized national welfare-assessment system (69). Overall, Chile has an extensive aquaculture regulatory architecture with explicit legal recognition of fish welfare and multiple complementary regulations addressing important welfare determinants. However, a gap remains between these legal and regulatory foundations and the systematic use of standardized animal-based indicators, welfare-specific intervention criteria, and verification of welfare outcomes. These characteristics describe the current degree of welfare operationalization but should not, by themselves, be interpreted as evidence of regulatory effectiveness, which requires independent evaluation based on measurable welfare outcomes.

### 5.2 Fish welfare legislation in Norway

Norway has developed a regulatory framework for farmed fish welfare that combines general animal-welfare legislation with aquaculture-specific operational requirements, systematic monitoring, and science-based welfare assessment (56, 70). The principal legal foundation is the Animal Welfare Act (2009) (Lov om dyrevelferd, Dyrevelferdsloven; LOV-2009-06-19-97), which explicitly includes fish within its scope, recognizes the intrinsic value of animals independently of their utility to humans, and establishes that animals must be treated well and protected from unnecessary stress and adverse impacts (71). The Act establishes a positive duty of care encompassing appropriate environmental conditions, feeding, supervision, and preventive management consistent with species-specific biological needs.

The Aquaculture Act (2005) provides the broader legal framework for aquaculture activities (72), while fish welfare is operationalized more explicitly through the Aquaculture Operation Regulations (73). These regulations translate general welfare principles into farm-level requirements concerning professional competence and training, contingency planning, facility and equipment design, water quality, stocking density, feeding and fasting, vaccination, crowding, pumping and handling, mortality, health inspections, slaughter, and record keeping. Producers are also required to maintain information concerning fish health and welfare, including health checks, injuries, treatments, diagnoses, and probable causes of disease and injury (74, 75). Transport is separately regulated through requirements concerning personnel competence, equipment, risk assessment, emergency planning, water quality, stocking density, handling, supervision, and care (76). These direct welfare requirements are complemented by sanitary, environmental, biosecurity, disease-control, and production regulations. Measures established under the Food Act address disease prevention, surveillance, biosecurity, and animal health, while specific regulations concerning infectious diseases and salmon lice can influence welfare both by reducing disease and parasite burdens and through the welfare risks associated with control procedures (77). Environmental and production-management instruments, including the national traffic-light system, provide an additional regulatory layer addressing biological and environmental risks associated with aquaculture production (78). Thus, the Norwegian framework combines direct welfare obligations with complementary regulation of major environmental, health, and husbandry determinants.

A particularly relevant development has been the incorporation of science-based welfare assessment into farm management. The FISHWELL project developed OWIs and Laboratory Welfare Indicators (LABWIs) for salmonids, contributing to the development of the LAKSVEL protocol for standardized welfare surveillance of Atlantic salmon in marine net pens (34, 79). Indicators including mortality, external injuries, fin condition, behavior, feeding activity, and environmental parameters provide a standardized and operational basis for welfare-risk assessment and management (80). However, the biological validation and regulatory applicability of individual indicators and associated thresholds may vary according to species, life stage, production context, and the welfare outcome considered. Importantly, the availability of standardized FISHWELL or related scoring systems should not be interpreted as evidence that all individual indicators, scores, or severity categories have been equivalently validated against underlying physiological or biological welfare states. External scoring systems necessarily simplify complex biological responses, and their interpretation should therefore be supported, where appropriate, by complementary physiological, health, or behavioral evidence.

More broadly, these developments should not be interpreted as demonstrating that Norwegian aquaculture has achieved a fully outcome-based welfare regime. Important challenges remain in the formal incorporation of animal-based outcomes into regulatory decision-making, while recurrent welfare concerns associated with mortality, crowding, pumping, delousing, handling, transport, and slaughter demonstrate that detailed regulation and scientific assessment tools do not necessarily translate into consistently improved welfare outcomes in practice (81). Thus, the Norwegian framework illustrates extensive operationalization of welfare requirements, but the effectiveness of these mechanisms must be evaluated independently through measurable welfare outcomes.

### 5.3 Fish welfare legislation in Scotland

Scotland has developed a distributed model of farmed fish welfare governance in which statutory animal-welfare obligations are complemented by aquaculture-specific legislation, fish-health and biosecurity regulations, industry codes of practice, technical standards, and science-based welfare-assessment tools (82). The principal statutory foundation is the Animal Health and Welfare (Scotland) Act 2006, which establishes a duty of care toward animals and requires responsible persons to ensure that their welfare needs are met (83). Farmed fish fall within this broader framework, although Scotland does not currently have a dedicated statutory welfare code covering farmed fish, meaning that implementation depends on complementary legislation, guidance, industry standards, and scientific evidence.

Specific production stages associated with elevated welfare risks are addressed through additional instruments. Transport is covered by the Welfare of Animals (Transport) (Scotland) Regulations 2006 (84), while slaughter and killing are governed by the Welfare of Animals at the Time of Killing (Scotland) Regulations 2012 and applicable requirements under Council Regulation (EC) No. 1099/2009 (85, 86). These provisions were further supported in 2025 by Scottish Government guidance specifically addressing welfare during salmon slaughter and related operations (87). Aquaculture production, fish health, biosecurity, containment, and farm management are regulated through additional sector-specific instruments, including the Aquaculture and Fisheries (Scotland) Acts of 2007 and 2013 and the Aquatic Animal Health (Scotland) Regulations 2009 (88–90). Although these instruments do not constitute a single comprehensive aquaculture-specific welfare regime, they regulate important welfare determinants, including disease and parasite management, biosecurity, containment, mortality management, and farm practices. The Aquaculture Code of Practice and the industry-developed Code of Good Practice for Scottish Finfish Aquaculture further translate regulatory and husbandry expectations into practical guidance concerning health, biosecurity, environmental management, handling, containment, and welfare (91, 92).

Scotland has also contributed to the development of science-based welfare assessment through its involvement in FISHWELL and the development of OWIs and LABWIs for farmed salmonids (34). In parallel, Scotland's Vision for Sustainable Aquaculture identifies improved aquaculture health and welfare and reduced cumulative mortality throughout the finfish production cycle as strategic priorities, while encouraging research, technological innovation, and improved welfare practices (82). However, these policy objectives do not themselves establish legally binding animal-based thresholds or standardized regulatory responses. Overall, Scotland represents a distributed model of welfare governance, in which general statutory welfare duties are operationalized through a combination of sector-specific legislation, government guidance, industry standards, and scientific assessment tools. However, the absence of a unified statutory mechanism linking standardized animal-based indicators to explicit intervention thresholds, corrective actions, and outcome verification limits the extent to which welfare outcomes are consistently operationalized at the regulatory level. As in the other jurisdictions examined, the existence and sophistication of welfare-related requirements should be distinguished from evidence demonstrating their effectiveness in improving measurable welfare outcomes.

### 5.4 Comparative analysis of fish welfare regulation in Chile, Norway and Scotland

The comparison of Chile, Norway, and Scotland reveals both common principles and important differences in how fish welfare is incorporated into aquaculture governance. All three jurisdictions recognize, either explicitly or through broader animal-welfare legislation, that farmed fish require protection from unnecessary suffering, and all regulate important welfare determinants through sanitary, environmental, biosecurity, transport, health, and production requirements. The principal differences therefore concern how these general welfare principles are translated into aquaculture-specific requirements, standardized monitoring procedures, animal-based indicators, intervention criteria, corrective actions, and regulatory oversight.

Importantly, regulatory operationalization and regulatory effectiveness represent distinct analytical dimensions. The existence of detailed welfare requirements, monitoring systems, or science-based assessment tools indicates the extent to which welfare principles have been translated into regulatory mechanisms, but does not by itself demonstrate that these mechanisms result in improved welfare outcomes. Accordingly, the present comparison focuses primarily on the mechanisms through which welfare is operationalized rather than assuming that greater regulatory detail necessarily results in better welfare in practice. Within this analytical framework, the three jurisdictions exhibit distinct regulatory architectures. Chile combines explicit legal recognition of fish welfare under the General Fisheries and Aquaculture Law (LGPA) (64) and the Animal Protection Law (68) with extensive sanitary, environmental, and production regulation through instruments such as RAMA, RESA, and REPLA (65–67). Article 13F of the LGPA establishes a general welfare mandate, while Article 32B of RESA provides a specific requirement concerning the prevention of unnecessary suffering during slaughter. In parallel, the regulatory system generates substantial information potentially relevant to welfare, including mortality, disease occurrence, stocking density, treatments, animal movements, and environmental conditions. However, these data are primarily interpreted within sanitary, environmental, epidemiological, or production-control frameworks rather than systematically integrated into a standardized welfare-assessment system. From an operationalization perspective, the principal gap therefore concerns the limited incorporation of standardized Animal-Based Measures (ABMs), Operational Welfare Indicators (OWIs), welfare-specific intervention criteria, and outcome-based verification into the national regulatory framework.

Norway is characterized by the direct incorporation of detailed welfare requirements into aquaculture operational regulation. The Animal Welfare Act establishes the general legal foundation (71), while aquaculture-specific regulations translate welfare principles into requirements concerning competence and training, water quality, stocking density, feeding, fasting, vaccination, handling, crowding, pumping, mortality, health inspections, slaughter, and record keeping (72, 73). Transport is additionally addressed through specific regulatory requirements (76), while the Food Act, disease and salmon-lice regulations, and environmental management mechanisms provide complementary regulation of important welfare determinants (77). These regulatory mechanisms are increasingly complemented by science-based welfare-assessment tools, including FISHWELL, OWIs, LABWIs, and LAKSVEL (34, 79), making welfare an observable, documentable, and increasingly measurable component of routine aquaculture management. However, these developments should not be interpreted as demonstrating that Norwegian aquaculture has achieved a fully outcome-based welfare regime. Important challenges remain in the formal incorporation of animal-based outcomes into regulatory decision-making, while recurrent welfare concerns associated with mortality, crowding, pumping, delousing, handling, transport, and slaughter show that detailed regulation and scientific assessment tools do not necessarily translate into consistently improved welfare outcomes in practice (81). Thus, the Norwegian framework illustrates extensive operationalization of welfare requirements, but the effectiveness of these mechanisms must be evaluated independently through measurable welfare outcomes.

Scotland, in contrast, can be characterized as a distributed model of welfare governance. The Animal Health and Welfare (Scotland) Act 2006 establishes a general duty of care (83), complemented by regulations addressing transport and killing (84, 85), while aquaculture-specific and aquatic-animal-health legislation regulates important welfare determinants such as disease, parasites, biosecurity, containment, escapes, and farm management (89). Rather than concentrating detailed operational fish-welfare requirements within a single aquaculture-specific regulatory instrument, implementation is distributed among statutory legislation, government guidance, sector-specific codes, technical standards, and industry instruments such as the Code of Good Practice for Scottish Finfish Aquaculture (91). The development of FISHWELL and associated OWIs and LABWIs provides an additional scientific basis for translating broad welfare obligations into practical welfare assessment (34). Scotland can therefore be characterized as a distributed governance model in which statutory welfare duties are complemented and operationalized through multiple regulatory, technical, and scientific mechanisms. Taken together, these differences indicate that the most informative comparison among the three jurisdictions is not a ranking of regulatory systems, but an assessment of the mechanisms through which welfare objectives are operationalized (Table 1). Direct comparison of regulatory effectiveness among Chile, Norway, and Scotland remains constrained by the limited availability of harmonized welfare-outcome data. Mortality, disease occurrence, injuries, treatment-related losses, behavioral alterations, and other animal-based outcomes are not necessarily defined, measured, or publicly reported using equivalent methodologies across jurisdictions. Consequently, differences in regulatory operationalization cannot be assumed to correspond directly to differences in welfare performance. Evaluating regulatory effectiveness requires independent analysis of measurable animal-based outcomes and evidence that regulatory interventions produce demonstrable improvements in the biological and behavioral state of farmed fish. Therefore, the comparative analysis identifies a broader regulatory challenge shared across jurisdictions: the need to connect legal welfare objectives with scientifically supported indicators, context-appropriate intervention thresholds, predefined corrective actions, outcome verification, regulatory accountability, and periodic scientific review. This distinction between the existence of welfare regulation, its operationalization, and its demonstrated effectiveness provides the rationale for the Evidence-Based Welfare Regulation (EBWR) framework proposed below.

**Table 1:** Comparative dimensions of fish welfare regulatory operationalization in Chile, Norway, and Scotland.

Comparative dimension	Chile	Norway	Scotland
Legal recognition of fish welfare	Explicit recognition within aquaculture legislation; complementary general animal-protection legislation	Explicit inclusion of fish within general animal-welfare legislation	Farmed fish covered by general statutory animal-welfare duties
Aquaculture-specific welfare requirements	Specific requirements exist, including prevention of unnecessary suffering during slaughter; broader welfare determinants mainly regulated through sanitary and environmental legislation	Detailed aquaculture-specific operational welfare requirements covering husbandry, handling, health, transport and slaughter	Welfare requirements distributed across animal-welfare, aquaculture, transport, killing, health and biosecurity instruments
Resource- and management-based indicators	Extensively incorporated through environmental, sanitary and production regulation	Extensively incorporated into aquaculture operational regulation and monitoring	Incorporated through legislation, guidance, technical standards and industry codes
Animal-Based Measures (ABMs)/OWIs	Limited standardized incorporation into national regulatory welfare assessment	OWIs increasingly incorporated into operational welfare assessment; FISHWELL/LAKSVEL provide standardized tools	FISHWELL and OWI/LABWI approaches provide scientific and operational assessment tools, but regulatory integration is distributed
Standardized welfare monitoring	Extensive sanitary/environmental monitoring, but limited integration into a welfare-specific assessment system	Structured health and welfare monitoring and record keeping	Monitoring occurs through multiple statutory, guidance and industry mechanisms
Welfare-specific intervention thresholds	Limited standardized animal-based thresholds	Some operational indicators and thresholds available; strength of biological validation varies among indicators	Some thresholds/guidance available, but not integrated within a unified statutory welfare framework
Corrective actions linked to welfare indicators	Limited formal linkage between ABMs/OWIs and predefined regulatory responses	Greater linkage between monitoring, management responses and regulatory requirements, although not uniformly outcome-based	Distributed among statutory requirements, guidance and industry standards
Auditing/regulatory oversight	Primarily sanitary, environmental and production-oriented oversight	Regulatory oversight includes explicit health and welfare requirements	Combination of statutory oversight, guidance, certification and industry mechanisms
Periodic scientific review/adaptation	Scientific evidence informs regulation, but no unified welfare-specific adaptive framework	Science-based welfare tools and regulatory mechanisms undergo continued development	Scientific assessment, policy guidance and industry standards undergo continued development
Evidence of measurable welfare outcomes	Available outcome information is not yet sufficiently harmonized to attribute welfare performance to regulatory mechanisms	Extensive outcome data are available, but persistent mortality and treatment-related welfare challenges demonstrate that operationalization does not necessarily ensure favorable outcomes	Welfare outcome information is available, but direct attribution of outcomes to the regulatory architecture remains limited

The table summarizes how fish welfare objectives are translated into regulatory and operational mechanisms across the three jurisdictions, including legal recognition, aquaculture-specific welfare requirements, welfare indicators, monitoring, decision thresholds, corrective actions, regulatory oversight, and scientific review.

## 6 From legislation to evidence-based welfare regulation

The evolution of fish welfare legislation has progressively expanded legal protection for farmed fish. Nevertheless, the existence of welfare provisions does not necessarily ensure acceptable welfare outcomes under commercial conditions (93). Regulations may establish duties to avoid unnecessary suffering, maintain appropriate environmental conditions, or apply suitable procedures during handling, transport, and slaughter, but these requirements can remain difficult to interpret, monitor, and enforce (94). The central challenge is therefore no longer merely to incorporate fish welfare into legislation, but to translate broad legal principles into measurable, scientifically supported, and operational standards and to verify whether their implementation effectively protects welfare. This transition can be conceptualized through Evidence-Based Welfare Regulation (EBWR). In this review, EBWR is defined as an integrative regulatory framework in which legal welfare objectives are operationalized through scientifically supported resource-, management-, and animal-based indicators, selected according to their biological validity, reliability, species- and life-stage specificity, feasibility, and strength of supporting evidence. These indicators are linked to standardized monitoring procedures, context-specific intervention thresholds or reference ranges, predefined corrective actions, outcome verification, regulatory accountability, and periodic scientific review. Under this approach, compliance is not determined exclusively by adherence to prescribed farming conditions or procedures, but also by evidence describing the biological and behavioral responses of the animals.

Importantly, EBWR does not propose that its individual components are novel. Animal-Based Measures (ABMs), Operational Welfare Indicators (OWIs), risk-based monitoring, intervention thresholds, corrective actions, and adaptive management are already established within animal-welfare science and aquaculture. Rather, the proposed contribution of EBWR lies in explicitly integrating these components within a regulatory feedback system that connects welfare objectives, measurement, decision criteria, corrective action, outcome verification, regulatory accountability, and scientific review. EBWR should therefore be understood as a regulatory integration framework rather than as a new welfare-assessment methodology (Table 2). Under this framework, legislation defines welfare objectives, science provides evidence-qualified indicators and reference values, producers monitor welfare risks and outcomes, and regulatory authorities verify compliance and require corrective action when necessary (Figure 1).

**Table 2:** Conceptual comparison of Evidence-Based Welfare Regulation (EBWR) with existing approaches to animal welfare assessment and regulatory management.

Approach	Primary focus	Main contribution	Limitation addressed by EBWR
Evidence-Based Animal Welfare	Scientific basis for welfare assessment	Evidence-supported indicators and decisions	Does not necessarily specify regulatory feedback
Outcome-based welfare assessment	Animal outcomes	Direct assessment of animal condition	Does not alone define regulatory responses
Risk-based regulation	Prioritization of welfare hazards	Prevention and proportional intervention	May not include biological outcome verification
Adaptive management	Iterative adjustment	Incorporation of new evidence	Not necessarily linked to enforceable welfare duties
EBWR	Regulatory integration	Links legal objectives → indicators → monitoring → decision criteria → corrective action → outcome verification → scientific review	Integrates these components within an accountable regulatory feedback architecture

The table summarizes the primary focus and contribution of evidence-based animal welfare, outcome-based welfare assessment, risk-based regulation, and adaptive management, and highlights how EBWR integrates elements of these approaches within a regulatory feedback architecture linking welfare objectives, evidence-qualified indicators, standardized monitoring, decision criteria, corrective actions, outcome verification, regulatory accountability, and periodic scientific review.

**Figure 1:**
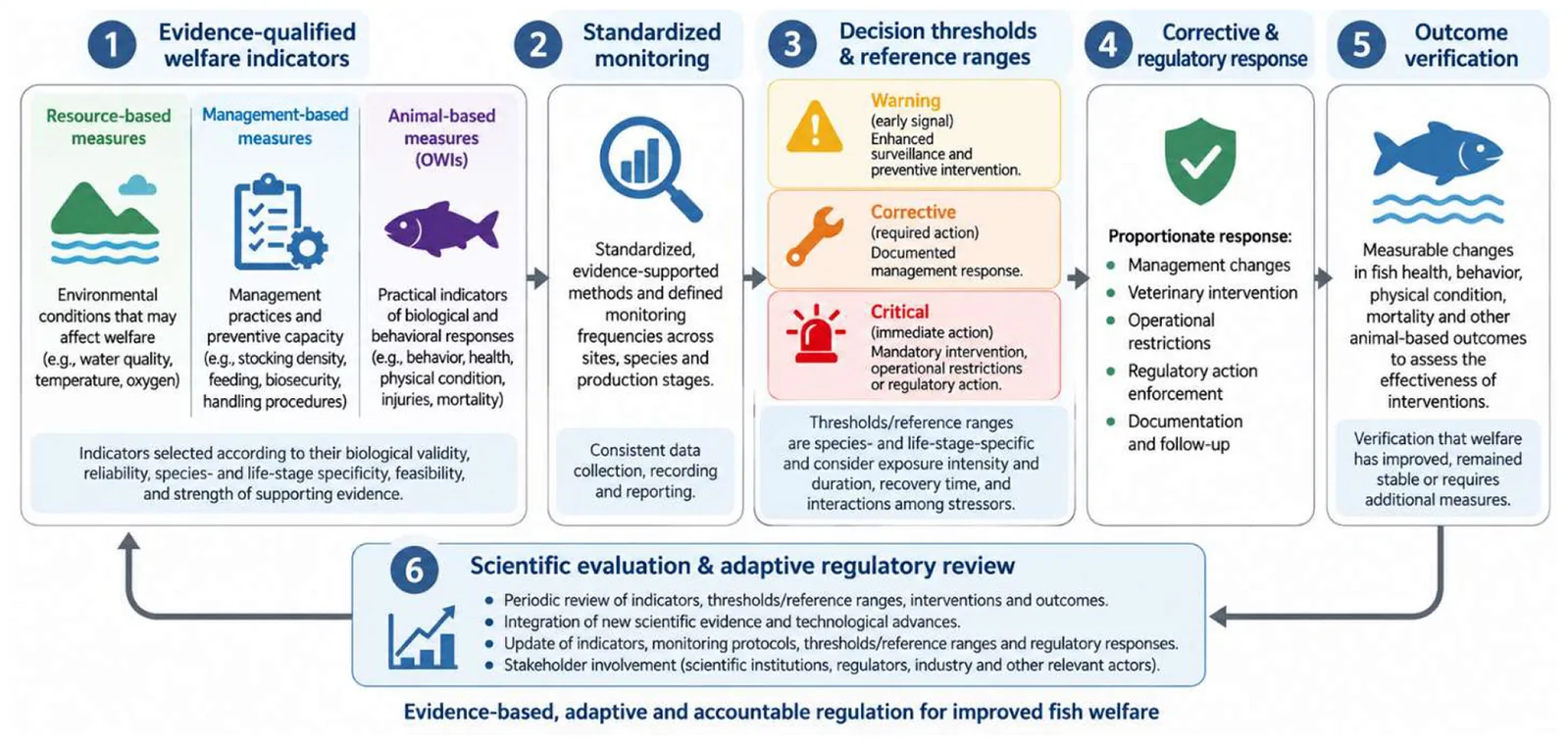
Conceptual framework for Evidence-Based Welfare Regulation (EBWR) in aquaculture. EBWR integrates resource-based, management-based, and animal-based measures (OWIs) within an adaptive regulatory feedback system. Evidence-qualified indicators are assessed through standardized monitoring and interpreted using species- and life-stage-specific decision thresholds or reference ranges. Exceedance of warning, corrective, or critical levels triggers proportionate management or regulatory responses. The effectiveness of these interventions is subsequently evaluated through measurable welfare outcomes. Scientific evaluation and adaptive regulatory review complete the feedback cycle by incorporating new evidence and technological advances to update indicators, monitoring protocols, decision criteria, and regulatory responses.

### 6.1 Integrating welfare indicators and biological evidence

EBWR should integrate, rather than replace, existing resource-, management-, and animal-based approaches. Environmental indicators identify potential welfare hazards, management indicators describe the practices and capacities used to prevent or mitigate them, and ABMs provide more direct evidence of how fish respond to farming conditions (5). OWIs can translate relevant biological responses into practical measures suitable for routine monitoring. Because fish welfare is multidimensional, individual indicators should not be interpreted in isolation. Regulatory assessment should therefore combine complementary environmental, behavioral, health, physiological, and production information within integrated indicator sets (95). However, operational feasibility should not be equated with biological validity. OWIs are practical proxies for welfare-related risks or biological responses and do not necessarily provide direct measures of the animal's internal welfare state. Their interpretation may depend on species, life stage, production environment, health status, individual history, sampling strategy, and observer variability. Furthermore, individual outcome-based measures may function as “iceberg indicators,” reflecting multiple underlying environmental, pathological, or management-related causes (70).

Consequently, the incorporation of an indicator into routine monitoring should not itself be interpreted as evidence that the indicator, or its associated threshold, has been sufficiently validated for regulatory decision-making (95). EBWR should therefore adopt a tiered assessment approach. Practical OWIs can provide a first level of routine welfare surveillance, while more detailed animal-based, physiological, veterinary, or laboratory-based indicators can be incorporated when deterioration is detected, when the cause of an adverse welfare signal remains uncertain, or when regulatory decisions require stronger biological evidence. Such an approach combines operational feasibility with biological verification while avoiding excessive reliance on individual external or categorical welfare scores. In Chile, this approach could build directly on the substantial sanitary, environmental, mortality, and production information already generated under existing regulations. The principal requirement would therefore not be to replace current monitoring systems, but to complement them with evidence-qualified animal-based indicators, appropriate reference values, standardized monitoring frequencies, and predefined decision and corrective-response criteria.

### 6.2 Risk-based, adaptive, and enforceable welfare regulation

EBWR should also be risk-based. Welfare risks differ substantially among production stages, procedures, environmental conditions, and facilities. High-risk events may include crowding, grading, pumping, vaccination, delousing, transport, prolonged fasting, exposure to poor water quality, thermal stress, disease outbreaks, mechanical injury, stunning, and slaughter. A risk-based system would require operators to identify foreseeable hazards, evaluate their probability and severity, implement preventive measures, and subsequently verify their effectiveness through measurable welfare outcomes (96). Such an approach should prioritize early detection rather than rely exclusively on reactive enforcement after mortality, disease, or severe injury has occurred. Changes in feeding behavior, swimming distribution, ventilation rate, schooling cohesion, surface activity, physical condition, or environmental variability could provide early-warning signals. When predefined limits are exceeded, increased monitoring and corrective action could be initiated before deterioration progresses to clinical disease or mortality. Scientific indicators, however, become meaningful regulatory tools only when connected to explicit decision rules.

Regulatory thresholds should not be regarded as universally applicable fixed values. Their biological relevance may depend on species, life stage, health status, environmental conditions, acclimation history, exposure intensity and duration, recovery time, and interactions among simultaneous stressors. Under commercial farming conditions, environmental, biological, and production-related factors may interact synergistically or antagonistically, meaning that thresholds derived from isolated single-factor studies may not adequately predict welfare outcomes. Where robust biological thresholds are unavailable, scientifically supported reference ranges or precautionary decision criteria may therefore be more appropriate than single numerical limits (70). Within this context, a tiered decision framework could distinguish warning, corrective, and critical thresholds. Warning thresholds would trigger enhanced surveillance and preventive intervention; corrective thresholds would require documented management responses; and critical thresholds could trigger mandatory veterinary intervention, restrictions on handling or production, emergency measures, or regulatory sanctions (97). Thresholds and reference ranges should be species- and life-stage-specific and consider not only the intensity of exposure but also its duration, frequency, recovery time, and interaction with other stressors (70).

Importantly, corrective action should not represent the endpoint of regulatory intervention. EBWR requires subsequent verification that the intervention produced the intended improvement in welfare indicators or animal-based outcomes. Failure to achieve improvement should trigger reassessment of the intervention, escalation of management responses, or regulatory action where appropriate. The regulatory sequence therefore becomes monitoring, welfare-risk detection, decision threshold, corrective action, outcome verification, and regulatory and scientific review. Regulation must simultaneously remain adaptive. Scientific understanding of fish welfare continues to evolve, and rigid legal thresholds may become outdated as knowledge advances. A flexible regulatory architecture could therefore establish broad legally binding welfare objectives while allowing technical standards, protocols, indicators, and thresholds to be periodically updated through scientific review. Elements of this approach can already be identified in existing aquaculture governance systems. For example, Norwegian regulation combines producer responsibility, preventive health management, professional competence, welfare monitoring, documentation, and corrective action (98). However, the presence of these individual components does not necessarily constitute a fully integrated EBWR system, particularly where links between animal-based outcomes, intervention thresholds, regulatory responses, and verification of subsequent welfare improvement remain incomplete. Effective implementation also requires accountability. Producers must understand which indicators are evaluated, how they are measured, and what consequences follow from non-compliance, while regulators require standardized protocols, trained inspectors, scientifically supported assessment tools, and reliable data. In Chile, existing institutional capacities in sanitary surveillance, traceability, inspection, and enforcement could provide a foundation for this transition, provided that welfare monitoring is supported by specific technical standards, personnel training, adequate resources, and clearly defined regulatory responses.

### 6.3 A pathway toward evidence-based welfare regulation in Chile

Based on the comparative analysis, the transition toward EBWR in Chile could follow four complementary and interconnected steps. First, fish welfare should be supported by a scientifically grounded and multidimensional regulatory definition that recognizes welfare as the physical and mental state of an animal in relation to the conditions in which it lives and dies (99). This definition should provide explicit regulatory objectives against which welfare protection can be evaluated. Second, species- and production-stage-specific welfare standards should be developed, initially for the main farmed salmonids and subsequently for other relevant teleost species. These standards should integrate resource-, management-, and animal-based indicators and establish appropriate monitoring frequencies (100). Selection of OWIs should be supported by transparent assessment of their biological validity, reliability, feasibility, species- and life-stage applicability, and overall strength of evidence rather than by operational convenience alone. Third, standardized welfare-assessment protocols should be implemented using evidence-qualified OWIs for routine farming and for high-risk procedures such as handling, crowding, grading, vaccination, treatments, transport, and slaughter (101). Monitoring results should be linked to scientifically supported and context-specific thresholds or reference ranges and predefined management responses. Welfare-management plans could complement these protocols by identifying foreseeable risks, preventive measures, responsibilities, emergency procedures, and escalating corrective actions. Finally, welfare outcomes and corrective interventions should be linked to regulatory oversight, outcome verification, and periodic scientific review (102). Scientific institutions, veterinary professionals, producers, and governmental agencies could contribute to evaluating whether interventions effectively improve welfare outcomes and to updating indicators, reference values, technical standards, and regulatory responses as new evidence becomes available.

This approach would allow Chile to build on its existing regulatory and institutional capacity rather than create a parallel welfare system. Under EBWR, legislation defines welfare objectives, science establishes evidence-qualified indicators and decision criteria, producers monitor and manage welfare, and authorities verify both compliance and outcomes. The regulatory focus therefore expands beyond demonstrating compliance with prescribed procedures to include verification that management practices and regulatory interventions effectively protect or improve measurable fish-welfare outcomes.

## 7 Future perspectives

The future development of Evidence-Based Welfare Regulation (EBWR) will depend on its capacity to respond to environmental change, technological innovation, species-specific biological variation, and evolving scientific knowledge. Future regulation should therefore remain adaptive while ensuring that emerging tools, indicators, and standards are supported by transparent evaluation of their biological validity, reliability, feasibility, and strength of evidence under commercial farming conditions. Importantly, future development should focus not only on expanding monitoring capacity, but also on determining whether regulatory interventions produce measurable improvements in welfare outcomes.

### 7.1 Climate change and emerging welfare risks

Climate change is likely to increase welfare risks through rising water temperatures, marine heatwaves, reduced oxygen availability, harmful algal blooms, altered salinity, ocean acidification, and changes in pathogen and parasite distributions (103). Because fish are ectothermic, these environmental changes can directly affect physiological performance, stress tolerance, disease susceptibility, and survival. These challenges reinforce the need for adaptive rather than static welfare regulation. Climate-related risk assessment, seasonal contingency planning, real-time environmental monitoring, and context-specific thresholds, reference ranges, or precautionary decision criteria should increasingly form part of welfare governance. Regulatory assessment should consider not only the magnitude of environmental events, but also their duration, frequency, cumulative effects, recovery periods, and interactions with other biological and production-related stressors (60). Consequently, regulatory limits derived under stable or single-factor conditions may require reassessment under increasingly variable environmental scenarios. Site authorization, spatial planning, and farming technologies may also need to account for projected future environmental conditions.

### 7.2 Emerging technologies for welfare monitoring

Precision aquaculture technologies may strengthen welfare monitoring by providing continuous information on both environmental conditions and fish responses (17). Environmental sensors, automated feeding systems, underwater imaging, acoustic monitoring, computer vision, and machine learning may facilitate early detection of alterations in behavior, feeding, swimming, body condition, lesions, parasite burden, or other indicators before severe welfare deterioration occurs (104). Molecular and physiological biomarkers may complement these approaches by identifying biological alterations before visible disease or mortality. However, their regulatory application remains limited by context dependence, sampling effects, and the lack of universal reference ranges (37). Their greatest value is therefore likely to emerge when incorporated into evidence-supported multilevel indicator panels combining behavioral, clinical, environmental, physiological, and production information. However, increased data availability should not itself be interpreted as improved welfare assessment. For regulatory purposes, technological outputs must be biologically interpretable and linked to clearly defined welfare risks, decision criteria, and management responses. Their regulatory value will therefore depend not only on measurement accuracy, but also on evidence that detected changes are meaningfully associated with relevant welfare outcomes.

Non-invasive monitoring approaches are particularly promising because they may allow repeated or continuous assessment while reducing handling-induced stress (105). Nevertheless, digital tools, algorithms, and biomarkers should support rather than replace direct observation, biological interpretation, veterinary assessment, and professional judgment. Regulatory adoption will require independent validation, standardized data-quality procedures, transparent interpretation criteria, assessment of performance under different production conditions, and appropriate data governance.

### 7.3 Harmonization and species-specific standards

International harmonization could help establish common welfare principles, terminology, assessment criteria, and minimum welfare objectives while reducing regulatory disparities among producing countries. International organizations can provide a shared scientific foundation, but harmonization should not imply identical regulatory requirements across jurisdictions (106). Species, production systems, environmental conditions, institutional capacities, and socioeconomic contexts differ substantially. Common welfare principles should therefore coexist with nationally appropriate implementation mechanisms and species-specific standards (107). Greater harmonization of indicator definitions, measurement protocols, and reporting practices would also improve the comparability of welfare-outcome data across jurisdictions and facilitate evaluation of regulatory effectiveness. For Chile, Norwegian and Scottish experiences can provide useful technical and regulatory references, but their application should be adapted to local species, farming conditions, environmental characteristics, and institutional capacities rather than based on direct legal transplantation.

### 7.4 Research, professional competence, and regulatory capacity

The transition toward EBWR will require a stronger interface between welfare science and regulatory implementation. Priority research areas include the biological validation and field reliability of species- and life-stage-specific indicators, establishment of context-appropriate thresholds or reference ranges, cumulative and multiple stressors, positive welfare, non-invasive monitoring methods, and the short- and long-term consequences of production procedures (108). Particular attention should be given to identifying which indicators are sufficiently robust for routine surveillance and which require complementary biological, physiological, or veterinary assessment before being used for regulatory decision-making.

Importantly, research should also evaluate the effectiveness of regulation itself. Welfare provisions should be assessed according to whether their implementation produces measurable improvements in animal-based outcomes under commercial conditions. Longitudinal studies, before-and-after evaluations of regulatory changes, and appropriately designed cross-jurisdictional comparisons could help identify implementation barriers and assess how prescriptive, performance-based, and incentive-based approaches influence welfare outcomes under different production contexts. Such evaluation is essential to distinguish regulatory operationalization from demonstrated regulatory effectiveness. Professional competence will also be essential. Farm personnel, veterinarians, managers, transport and slaughter operators, and regulators should be capable of identifying early welfare deterioration, interpreting relevant indicators, implementing corrective actions, and recognizing when production procedures should be modified or stopped (109). Transparency, auditing, benchmarking, and private certification may complement public regulation, but they should not replace governmental oversight (110). Regulatory authorities will therefore require not only appropriate legal mandates, but also trained personnel, standardized assessment protocols, reliable data systems, and sufficient technical capacity to interpret welfare information and verify the effectiveness of corrective interventions.

## 8 Conclusions

The comparative analysis of Chile, Norway, and Scotland demonstrates that fish welfare governance extends beyond the formal recognition of welfare in legislation and depends on how legal objectives are translated into operational mechanisms. The three jurisdictions differ in the integration of welfare-specific requirements, monitoring systems, animal-based indicators, decision thresholds, corrective actions, and regulatory oversight. However, greater regulatory operationalization should not be interpreted as evidence of greater regulatory effectiveness. Demonstrating effectiveness requires independent evaluation of measurable welfare outcomes under commercial farming conditions. Evidence-Based Welfare Regulation provides an integrative framework for connecting these elements within a regulatory feedback system based on welfare objectives, evidence-qualified indicators, standardized monitoring, context-specific thresholds or reference ranges, corrective actions, outcome verification, regulatory accountability, and adaptive scientific review. Its proposed contribution lies not in the novelty of these individual components, many of which are already established in welfare science and aquaculture, but in their systematic integration within an accountable regulatory architecture.

For Chile, this transition could build on existing sanitary, environmental, monitoring, and institutional capacities rather than require the creation of a parallel regulatory system. Species- and production-stage-specific standards, evidence-qualified OWIs, standardized assessment protocols, context-appropriate decision criteria, professional competence, and outcome verification could progressively strengthen the incorporation of welfare into existing governance structures. Norwegian and Scottish experiences provide useful regulatory and technical references, but their application should remain adapted to local species, production conditions, available scientific evidence, and national institutional capacities. Ultimately, an evidence-based fish welfare regulatory system should be evaluated not by the number or complexity of its welfare provisions, but by its capacity to detect welfare risks, trigger proportionate interventions, verify their effectiveness through measurable welfare outcomes, and adapt as scientific evidence, environmental conditions, and production technologies evolve.
